# Associations Between Carotenoid Status, Visual Outcomes and Cognitive Metrics in Children: A Scoping Review

**DOI:** 10.3390/nu18132147

**Published:** 2026-07-02

**Authors:** Payal Sangani, Pinakin Gunvant Davey

**Affiliations:** College of Optometry, Western University of Health Sciences, Pomona, CA 91766, USA; psangani@westernu.edu

**Keywords:** carotenoids, cognition, macular pigment, lutein, xanthophyll, human milk, children, visual function, cognitive function

## Abstract

Background: Carotenoid components have a role in maintaining eye and brain development and function in children. Objectives: This scoping review collates the current knowledge about the benefits of carotenoids in improving visual and cognitive function outcomes in children. Design: This review includes observational and interventional published studies to date (January 2026) through a literature search on PubMed, Web of Science, Embase, Mendeley, and Google Scholar database platforms. We have described and measured carotenoid status in diet, serum, skin, and human milk in relation to eye and cognitive development and function in children. Results: Human body carotenoids, α-carotene, β-carotene, lycopene, cryptoxanthin, lutein, and zeaxanthin, have been detected in significant amounts in the brain, whereas the xanthophylls lutein, zeaxanthin, and meso-zeaxanthin, are found in the eyes. Lutein, zeaxanthin, and retinoids in the ocular tissue are responsible for visual function and have been associated with visual neurocognitive skills in children. In addition, lutein, zeaxanthin, and β-carotene have been associated with cognitive function tasks in infants and children. Conclusions: This review shows emerging evidence on the benefits of carotenoids in children, primarily from cross-sectional studies and longitudinal cohort studies. We found a paucity of carotenoid intervention and controlled trials for direct carotenoid exposure and its relative effect on neurocognitive function and visual milestone development during early life. Additionally, further long-term studies are required to confirm carotenoid exposure in early life and its benefits to eye health and cognitive skills outcomes in later life.

## 1. Introduction

Carotenoids are biological fat-soluble pigments that naturally occur in plants, bacteria, algae, fungi, and archaea in color shades of red, yellow, or orange [1]. These micronutrients can be ingested through dietary food sources or nutritional supplements. The consumed carotenoids depots are found in adipose tissue, liver, brain, and eye tissues, while circulating forms can be found in human milk and serum. An individual’s carotenoid status can be assessed using dietary food records, skin resonance Raman spectroscopy (RRS), and blood serum analysis. Ocular levels in the form of xanthophylls in the retina are measurable with direct (objective) and indirect (psychophysical) technologies that measure macular pigment [2,3,4]. Retinal carotenoid forms support visual and biological functions [5,6,7].

**Figure 1:**
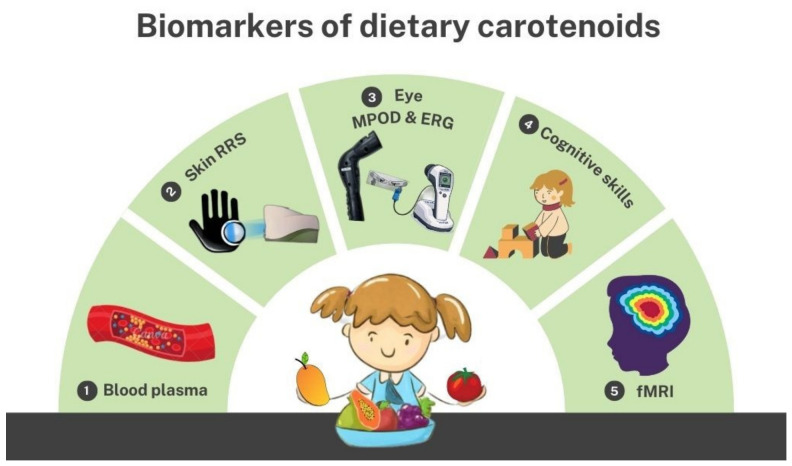
Dietary carotenoid reservoirs in humans are measurable through (1) Blood serum and plasma, (2) Skin Resonance Raman Spectroscopy (RRS), (3) Macular Pigment Optical Density (MPOD), and Electroretinography (ERG) in the eyes, (4) Cognitive skill tests, and (5) functional Magnetic Resonance Imaging (fMRI). This diagram was created by the authors using Canva and Google ImageFX AI (2025) tools.

Lutein and β-carotene are highly distributed in blood plasma, human milk, and brain tissues compared to other forms of carotenoids [8,9,10]. β-carotene is not found in its original form in the eye, but it is converted to circulatory retinoid forms, and then to 11-cis-retinal at the retinal level, which contributes to the formation of rhodopsin and iodopsin in rods and cones, respectively. Lutein is highly concentrated in the occipital region of the brain and in the macular region of the eye in humans and primates [11]. Lutein and β-carotene have been detected in the cadaver brain tissue of older individuals and were linked to their previously recorded cognitive ability [10]. It is important to note that ocular xanthophyll levels are also closely correlated with the levels of lutein and zeaxanthin in the occipital cortex [11]. Quite appropriately, the non-invasive nature of measuring levels of macular pigment via optical techniques, known as macular pigment optical density (MPOD), makes it an extremely useful surrogate biomarker of carotenoid levels in the brain and cognitive functions in adults. There is an increase in its use as a biomarker in children; however, additional research using this technology is needed before its exact hierarchical position in the list of biomarkers is established (see Figure 1).

The levels of carotenoids in individuals are dependent on dietary intake, which varies with demographics and the dietary habits of an individual. Additionally, their bioavailability is substantially influenced by dietary fat co-consumption, body mass composition, genetic factors, systemic health status, and age. The serum levels of carotenoids and MPOD are indeed modifiable either by increasing specific dietary intake or by supplementation with nutraceuticals [12,13]. Human milk and alternative infant milk formulas are primary sources of diet in the early months of life. It is reported that dietary carotenoids are scarce and their requirements are often unmet in American women of childbearing age [14,15]. This is especially evident during the third trimester, when compared to healthy non-pregnant women [16,17]. Additionally, the use of infant formula, as an alternative to human milk under some circumstances, has become a common practice worldwide [18,19]. The United Nations recently reported [20] that these formulas are not evaluated and approved scientifically and has questioned their recommendation as an alternative to mother’s milk during the critical period of development in children [19,21]. Currently available and marketed infant formulas include carotenoids; however, it has an unlikely concentration, and the forms of carotenoids do not biomimic human milk [9,22,23]. Given that these infant formulas do not effectively match the levels of carotenoids in human milk, especially in terms of the availability of bioactives and metabolites, it is not surprising that, while they do improve blood plasma levels, they are relatively less bioavailable than human milk [9,24,25]. In this review, we investigated the available evidence on the short-term effects and safety of carotenoid-fortified milk formulas, particularly lutein and zeaxanthin supplementation, on the eyes and cognitive function in children. Although both lutein and zeaxanthin are in a class of carotenoids often graded as GRAS (Generally Recognized As Safe) and their safety dosage in adults has been described elsewhere [26,27,28], their optimal daily requirement remains unknown, especially for children’s milestone development.

Micronutrient deficiency has become a global concern due to the dietary paradigm shift, with processed and ultra-processed food becoming common in the diet [29]. Such food items have been processed with artificial compounds for prolonged shelf life, safe usage, and attractive, colorful looks, but they lack biochemically active essential micronutrients [30,31]. One of the United Nations Sustainable Development Goals (SDGs) is to end micronutrient deficiencies by 2030 and enhance well-being [32]. This can be achieved by the profound inclusion of micronutrients in the diet or micronutrient fortification, as suggested by the World Health Organization (WHO) and the United States Food and Drug Administration (USFDA). In terms of carotenoids, there is a deficiency of dietary β-carotene, which transforms into retinol, a form of vitamin A, along with iron and zinc, in preschoolers. The deficiency of other carotenoids remains unclear due to a limited number of studies on optimal levels in healthy individuals and their function. However, it must be noted that micronutrient deficiency is most common in children and lactating women [33].

Carotenoid measurement and screening protocols for preterm infants, lactating mothers, and young children are not yet part of routine care in general pediatric or ophthalmology clinics. However, carotenoid deficiency from birth through early childhood may contribute to adverse outcomes related to skin health, metabolic function, and vision. Although their roles in preventing night blindness, skin cancer, age-related cognitive decline, and macular degeneration are well established, emerging evidence also suggests benefits for visual function and potential associations with cognitive performance. These findings indicate that dietary carotenoid intake may be clinically beneficial for children and could be particularly important for high-risk groups, including those born prematurely, formula-fed infants, children with known vitamin A deficiency, and those with obesity.

Developmental milestones are influenced by multiple factors, including overall diet, socioeconomic status, education, genetics, and general health. Current evidence linking carotenoids to developmental outcomes is largely associative, leaving causation unclear. It remains uncertain whether carotenoids directly improve developmental outcomes, which specific visual, cognitive, or developmental measures are affected, and at which developmental stages these effects occur. We indeed acknowledge that there has been a tremendous increase in research on the importance of carotenoids in developing individuals, but the interventional body literature is not sufficient for a systematic review or meta-analysis. For these reasons, this scoping review was designed to summarize existing evidence and identify gaps in knowledge that may guide future research on the role of carotenoids in children’s visual and cognitive development. This review aims to highlight and consolidate the status of carotenoids in children, along with their importance during the developmental years. This review includes the association of carotenoid measures in relation to developmental years, particularly cognitive skills and performance, in children. Further, it explores the evidence of the importance of carotenoids in developmental cognition milestones in children.

## 2. Materials and Methods

A comprehensive review of the literature was conducted through an electronic search of publications on PubMed, Web of Science, Google Scholar, Embase, and Cochrane Library databases. The electronic search was performed using a combination of keywords using Boolean operators (AND, OR): carotenoids, lutein, human milk, infant formula, infant, children, visual function, MPOD, macular pigment, cognition. We reviewed all the available literature between 1950 and January 2026. Studies were screened and selected based on the PICO framework as follows: Population (P)—Lactating mothers, preterm, infants and children of up to 18 years of age, which have studied the effects of breastfed or infant formulas; Intervention/Exposure (I)—Dietary intake of carotenoids, lutein through breastfeeding, donor human milk, fortified infant formula, or supplementation or have estimated carotenoid levels through dietary intake, measurement in serum, or at tissue level; Comparator (C)—Difference in carotenoids intake, carotenoid-fortified infant formula versus breastfeeding versus unfortified milk formula or control/placebo group; Outcomes (O)—Measured visual function, visual learning skills, cognitive skills, neurodevelopmental outcomes, improvement in carotenoid levels, measure of macular pigment optical density, skin carotenoids, and serum carotenoid levels.

A comprehensive literature search was conducted to identify relevant studies examining the relationship of carotenoids with visual and cognitive function in children. The search was based on predefined keywords: “Children,” “Carotenoids,” “Lutein”, “Vision,” and “cognition,” and they were searched in combination as below:

1. Child

(((((((child[tw]) OR (children[tw])) OR (kid[tw])) OR (infant[tw])) OR (toddler[tw])) OR (school[tw])) OR (infant, premature[tw])) OR (congenital[tw])

2. Carotenoids source

(((((((((((carotenoid[tw]) OR (carotenoids[tw])) OR (lutein[tw])) OR (lycopene[tw])) OR (beta Carotene[tw])) OR (Infant Formula[tw])) OR (Breast Feeding[tw])) OR (human milk[tw])) OR (breast milk[tw])) OR (vitamin a[tw])) OR (macular pigment[tw])) OR (retinopathy of prematurity[tw])

3. Vision outcome

((((((vision[tw]) OR (visual function[tw])) OR (macular pigment[tw])) OR (myopia[tw])) OR (hyperopia[tw])) OR (amblyopia[tw])) OR (strabismus[tw])

4. Cognitive outcome

(((cognition[tw]) OR (cognitive function[tw]) OR (brain[tw])) OR (memory[tw]) OR (learning[tw]))

For each keyword, corresponding MeSH terms and text word variations were used to ensure a broad and inclusive search. We included the studies that were published in peer-reviewed journals in English with full-text availability and were original research articles and clinical trials. The scoping review was registered at PROSPERO 2026 CRD420261420121.

### 2.1. Eligibility Criteria

Only articles published in English were included. Only human studies were considered to provide a comprehensive overview of the potential effects of carotenoids on visual and cognitive outcomes in children. No publication year restrictions were applied.

Studies were included if they met the following conditions:

1. Investigated the association between carotenoids and visual and cognitive outcomes in children;
2. Assessed relevant biomarkers or retinal outcomes;
3. Were original research articles (interventional, observational, or experimental).

Reviews, editorials, conference abstracts, and non-original studies were excluded.

### 2.2. Study Selection

**Figure 2:**
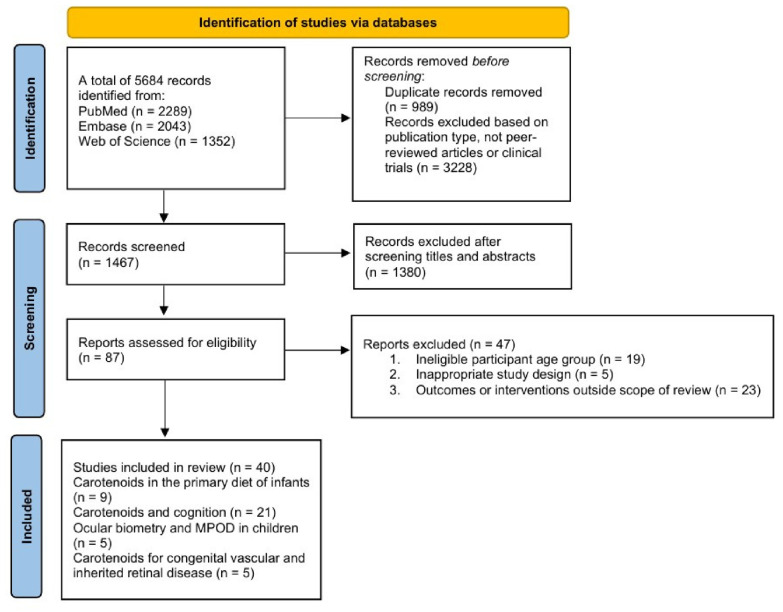
PRISMA flow diagram for selection of studies that investigate carotenoids and their association with visual and cognitive function outcomes in children.

Figure 2 provides the PRISMA Flow Chart for the scoping review on carotenoid status, visual outcomes, and cognitive metrics in children. Following screening of titles and abstracts, irrelevant studies were excluded as per the methods mentioned above, resulting in 40 articles that met the inclusion criteria and were included in the scoping review. These studies formed the basis for data extraction and analysis. Animal studies, or evaluation of interventions on animals, were not included in the primary review framework and were only utilized for supporting and explaining mechanisms.

Our objective for this review was to evaluate the following: (1) the presence of carotenoids reservoir in the eye and brain tissue and delineate the role of carotenoids in infants’ and children’s eye and brain development; (2) the serum and tissue level proportion of carotenoids in pregnant women, infants, and children and its interrelationship with the children’s neurocognitive function; (3) the preventative and therapeutic role of carotenoids in refractive error and congenital retinal conditions. The Supplementary Material Table S1 provides the list of studies included in the scoping review. 

Google ImageFX 2025 and Canva 2025 tool was utilized to produce image icons for Figure 1 via writing the appropriate prompt “generate a doodle picture of a girl kid eating fruits”.

## 3. Carotenoids in the Primary Diet of Infants

Human milk contains carotenes and xanthophylls, which are essential micronutrients for infants. Additionally, human breast milk lipids consist of fat-soluble enzymes that are essential for carotenoid metabolism and transport to various parts of the body, including the eyes and the brain. Carotenoids serve many fundamental roles in the physiology of growing children. They are known for their antioxidant, anti-inflammatory, and immune-boosting properties [34,35]. Lutein is abundantly concentrated in the brain region compared to other dietary carotenoids, especially in the occipital region. The occipital region of the brain is connected to the eyes and serves as an analyzer of visual stimulation and cognitive functions and is also the region responsible for vision and perceptual learning functions. The xanthophylls in the retina are a collection of lutein, zeaxanthin, and its isomer meso-zeaxanthin that are deposited throughout the retina in different concentrations. In the central retina, the xanthophyll-carotenoids are highly concentrated, forming a dense yellow spot, or the macular pigment. The xanthophylls in the macular region serve numerous functions, including antioxidant, anti-inflammatory, and enabling visual processing. At the embryonic and infant stages of life, these structures are still in critical developmental period and remain so until the age of six. Lutein and oxylutein were found in human embryonic eyes at an elevated concentration between 12 and 22 weeks [36], during the prenatal period of the second trimester. Biochemical analysis and autopsy results of human embryonic eyes have shown the presence of lutein and oxylutein in the vitreous, retinal pigment epithelium, lens, retina, ciliary body, and iris from the first trimester at 15 weeks of gestation. Interestingly, lutein concentration in the vitreous is undetectable during the second trimester when the retina starts developing new blood vessels and begins to form the fovea. During this period, carotenoids form a border between the retina and vitreous [36], thus postulating a role of lutein in the formation of the fovea and foveal avascular zone during infancy [37]. The MPOD levels are lower in prematurely born infants than in healthy full-term newborns [38]. In prematurely born infants, MPOD is undetectable at the retinal level [39] with a Retcam-2 imaging device, but minimally detectable with advanced retinal imaging with a Retcam-3 device [40,41]. Children in their initial years of life acquire carotenoids from their mothers. During embryonic development, maternal serum is the reservoir and the source of carotenoids. During infancy, the exclusive source of carotenoids is human milk or infant formula. It is not surprising but incredibly important to note that children’s early development is causally linked to maternal health during pregnancy. Thus, maternal carotenoid levels in serum, skin, and retina are associated with their children’s well-being and contribute to the functional development of their offspring through direct serum transport prenatally and breast milk supplementation in infants [42,43].

### 3.1. Carotenoids and Preterm Infants

Several studies in the last decades have reported low carotenoid levels in the plasma of preterm-born infants, in their mothers, and in their milk compared to full-term-born infants and their mothers [43,44,45]. Recently, human milk mapping of mothers with preterm and full-term babies in Switzerland revealed that mothers with preterm babies have a low carotenoid content in their breast milk compared to mothers with full-term newborns [44]. This would suggest that mothers may require additional nutritional supplements or carotenoid-fortified infant formulas to meet the optimal micronutrient amount if a child is born preterm.

Recent placebo-controlled RCTs completed among dyads of full-term-born children and mothers indicate that prenatal exposure to 10 mg lutein and 2 mg zeaxanthin for 9 months contributes to improved maternal serum, skin, and retinal carotenoid levels compared to placebo controls. This, in turn, results in a significant increase in levels of carotenoids in infant skin and in umbilical cord blood, with a 20% increase in MPOD and early maturation of the central retinal structures [41,42]. The results of these studies are the first of their kind, and additional research is definitely warranted prior to broad applications. The results of these studies may have numerous clinical implications and suggest the need to monitor carotenoid levels during the second and third trimesters of pregnancy and recommend supplementing with at least 10 mg of lutein and 2 mg of zeaxanthin [41,42] to meet the optimal nutritional needs of women with carotenoid deficiency. Studies have also shown the effect of postnatal carotenoid supplementation on cognitive skills in later years, which we discuss in a later section.

### 3.2. Human Milk Versus Carotenoid-Fortified Milk Formula

Lutein and other forms of carotenoids from human milk appear to be more bioavailable and effective at raising infant serum carotenoid levels than fortified infant formulas [46,47]. Human milk consists of biological macronutrients, bioactives and metabolites, such as phospholipids, milk fat globules (polar lipids), organonitrogen compounds, and amino acids, that make them more bio accessible and bioavailable at the tissue level [48,49,50,51]. One study evaluated the bioavailability of carotenoids from commercial infant supplementation formula and breast milk in rhesus macaques. The outcome of the study showed that lutein and zeaxanthin from breast milk were highly concentrated in serum and more bioavailable to the macular region of the retina and occipital region of the brain tissues compared to supplemented infant formula [25]. Here, it has to be noted that the zeaxanthin amount in rhesus macaque breast milk differed markedly from the experimental formulas. Zeaxanthin concentration was approximately five-fold higher in breast milk than in the supplemented formula. These findings suggest that differences in the accumulation of the brain carotenoids are a function of both (1) quantitative differences in supplemented milk formula and breast milk content and (2) superior bioavailability from breast milk [25]. Here, the presence of other carotenoids along with zeaxanthin in the infant formula may hinder the overall absorption of carotenoids in the baby macaques, as multiple carotenoids compete for absorption. These findings from animal studies are exciting indeed, but we need to temper our excitement, as they cannot be directly applied to humans. Thus, studies are needed to establish the optimal ratio of carotenoids and xanthophylls-to-lipoproteins relative to human milk contents so that formulas can meet the developmental nutritive needs of infants’ eyes and developing brains.

Similarly, in humans, when fortified infant formula (lutein concentrations of 68.7 and 211 mcg/100 g) was fed to preterm babies for 40 weeks, it resulted in a significant increase in plasma levels of lutein, β-carotene, and lycopene. This, in turn, showed a reduction in C-reactive protein levels compared to the control group. Additionally, the improvement in the plasma lutein levels correlated positively with rod photoreceptor sensitivity, as measured using a full field electroretinogram [52].

Another primate study showed early maturation of cortical regions as assessed by structural changes and functional magnetic resonance imaging evaluations at 6 months from birth when supplemented with carotenoids [53]. The study showed improved connection of memory and motor functions at four months and visual motor and auditory motor function connection at six months in the carotenoid-supplemented group when compared to the breast milk-fed group of infant macaques that had no supplementation of carotenoids. These animal studies provide good insight into possible mechanisms and outcomes; however, these results should likely be interpreted with caution and are not directly applicable to humans. The effect of carotenoid-fortified infant formula has been primarily assessed in association with serum biomarkers of infants, with limited evidence for retinal and cognitive outcomes. There is a lack of studies that have looked at carotenoid status or MPOD in children who have only been fed with infant formulas and not mother’s milk. This will be a difficult situation to assess, as nutritionally breast milk is considered the best for infants. Furthermore, the effect of commercially available fortified formula supplementation is yet to be studied among preterm-born children with a risk of secondary eye and cognitive complications.

### 3.3. Role of Early Exposure to Carotenoids in Cognition and Visual Function in Later Life

**Figure 3:**
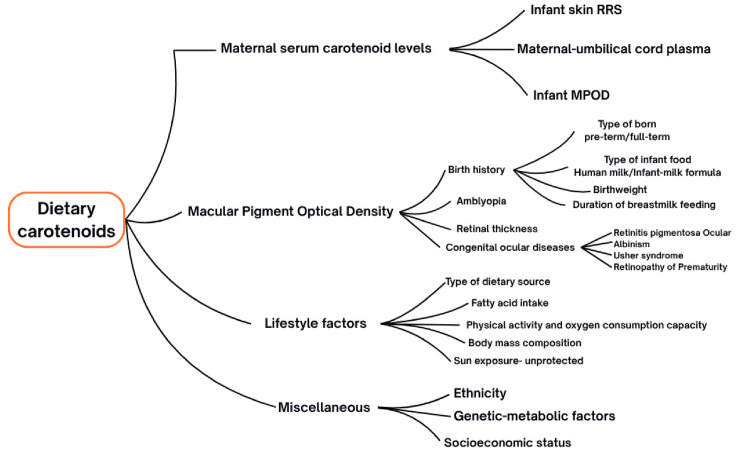
Describes the potential factors that influence the local distribution of dietary carotenoids in children.

The first six years of life are critical for achieving visual and cognitive development and are linked to complex environmental exposure during the “critical developmental period”. Figure 3 shows the complex interactions of dietary carotenoids. Explanation of all the factors that may determine the distribution of carotenoids in infants is beyond the scope of this review article, but a short discussion is essential. Briefly, carotenoids cannot be synthesized de novo as they are consumed via diet. This forms the serum levels, which subsequently form MPOD in the retina [54]. The serum levels, biological invasive methods, and MPOD can be used as biomarkers [2,55]. The levels of carotenoids vary under various conditions, such as disease states and premature infants, etc. [56]. Additionally, numerous factors influence the levels of carotenoids. Lifestyle factors, like sources of carotenoids, sun exposure of individuals, co-intake of fats, and adiposity, all influence the levels of carotenoids in the body [57]. Additionally, genetic or ethnic variations in carotenoid levels have also been reported [58,59,60]. It should be noted that most understanding of these mechanisms comes from adult studies, and further research is needed on MPOD to establish its status as a neural biomarker in the pediatric population.

The critical developmental period to achieve these milestones varies among individuals. Carotenoid exposure in early life through maternal serum and human milk may have an association with cognitive function, as these carotenoids may work as the primary concentrated elements of the tissue during its establishment and development, in addition to the circulatory forms of carotenoids seen in later life. Among school-aged children, longer duration of exclusive breastfeeding showed high skin carotenoid levels in later childhood, and the duration of breastfeeding had a significantly inverse association with BMI [61]. Children with medium to high adherence to a Mediterranean diet reported no significant association between MPOD and visual function parameters, i.e., visual acuity and contrast sensitivity [62]. A study in adults aged 40–65 showed that the human milk-exposed cohort seems to have a higher level of MPOD compared to unknown milk formula-fed participants (mean MPOD −0.54 ± 0.07 vs. mean MPOD = 0.38 ± 0.06) [63]. This related area of research requires additional exploration. Future studies, focusing on the role of nutrition and cognitive activity exposure during critical developmental periods, may help in understanding the interrelationship of the eye and brain carotenoids and their influence on delays in developmental milestones. The role of carotenoids in visual milestone development has not been linearly investigated with long-term controlled trials with nutrition and cognitive interventions in literature. Establishing these could lead to a better understanding of how carotenoids could potentially contribute to changes in visual sensory function, along with anatomical maturation of the retina during early childhood.

Most studies related to the assessment of eye and brain biomarkers of development are performed in the American region, where carotenoid concentration, particularly lutein and zeaxanthin, is at low levels compared to other countries. The study of these biomarkers in other regions or multi-ethnicity group is particularly essential.

## 4. Carotenoids and Cognition

Over the last decade, there has been a growing interest in supplementing and fortifying children’s diets with carotenoids to overcome the challenge of micronutrient deficiency and improve cognitive skills and academic performance (see Table 1). Longitudinal cohort studies [64,65,66,67] have shown that for infants, human milk is the primary nutrient source, and its beta-carotene and lutein components contribute to the development of recognition, memory, and psychomotor development in the first six months of life [64,65]. In toddlerhood, a study among ethnic groups in Singapore showed that children’s skin carotenoids and the history of maternal beta-cryptoxanthin levels are related to cognitive and motor skill development, as assessed through the BSID-II test [66].

Preschool-aged children who were fed with human milk for more than three months exhibited better performance on the Pictorial Vocabulary Test (PPVT); however, the preschoolers’ serum lutein levels in the study did not reveal any association with other assessed cognitive parameters [66]. A recent study by Mahmassani et al. [67] in the United States observed increased cognitive and behavioral scores on PVT-III tasks in individuals with high lutein and zeaxanthin dietary intake [67]. Various cross-sectional studies [60,68,69,70,71,72,73] have shown associations between carotenoid status and cognition. Yen et al. [68] reported a positive association between skin carotenoid levels and general intellectual ability. Skin carotenoid levels were not associated with the other cognitive skills, academic performance (ECADTM), and expressive language tasks that were assessed. Although it is not possible to ascertain causation in a cross-sectional study, one could speculate that preschoolers’ general intellectual ability may develop prior to competency for academic performance and language skills, which may follow in later years. *BCO1* and *CD36*, alleles that are involved in carotenoid metabolism, are linked to the MPOD level of children [60,69].

The KTEA-II (Kaufman Test of Educational Achievement) test measures academic achievement in children. Children’s performance on this test has been reported to be positively linked to MPOD levels and dietary intake [60], while high MPOD is associated with cognitive processing for incongruent tasks on Event-Related Potential measures [41]. Additionally, the other measures of cognition, including Woodcock–Johnson III tasks (intellectual ability, verbal ability, cognitive efficiency, executive processes, spatial relation, visual/auditory learning), hippocampal memory, and IQ have also shown a positive association with MPOD in this age group [70,71,72]. A recent study has shown a positive correlation between the skin carotenoid scores obtained using reflection spectroscopy and reasoning and math skills [73].

An RCT in children of Asian Indian descent showed that lutein and zeaxanthin supplementation for six months resulted in an improvement in the serum neuroprotective factor BDNF and various cognitive performance components like attention, focus, episodic memory, visual learning, visuospatial working memory, and processing speed. Subsequently, the participants showed an increase in serum levels of xanthophylls and MPOD [74]. Although it appears that performance testing with BSID, KABC, PPVT, and Woodcock–Johnson tests show improvement, it must be acknowledged that these tools assess different domains and different age groups and are not directly comparable with each other.

The current literature highlights the importance of four carotenoid biomarker tests that may directly or indirectly relate to cognitive function development in children. However, it should be noted that the evidence is still too limited and scattered to conclude the causation and the true effects of carotenoid forms on cognition components throughout the critical period of development. Published research often utilizes variable methodology as the “gold” standard to assess cognitive and carotenoid function biomarkers across the age groups, and it is not established (see Table 1).

**Table 1:** Studies reported the impact of blood plasma, ocular, skin, and dietary carotenoids on cognitive development and academic performance.

Study Author, Year	Age	Cognitive Function Test Battery	Assessed Carotenoid Components				Study Outcomes
			MPOD	Serum Carotenoids	Skin Carotenoids	Dietary Intake	
Cheatham and Sheppard, 2015 [64] ★★ (USA)	Infants, 6 months (n = 55)	Recognition memory: ERP and eye movements	-	-	-	Human milk Lutein levels	Human milk lutein levels were significantly associated with recognition memory (*p* < 0.05).
Zielinska, 2019 [65] ★★ (Poland)	Infants, 6 months (n = 39)	Psychomotor development: Children’s Development Scale (DSR)	-	-	-	Human milk carotenoid contents were assessed	Human milk β-carotene content related to psychomotor development among infants (β = 0.348 *).
Rosok, 2024 [75] ★ (USA)	12–18 months (n = 45)	BSID-IV: Cognitive, language, and motor skills MMN with an auditory oddball task, VEPs with a pattern reversal task	-	-	Veggie Meter	-	Skin carotenoids were only associated with cognitive skills (β = 0.24 *). MMN and VEPs outcomes were not associated with skin carotenoid scores.
Lai, 2020 [66] ★★ (Singapore)	Age: 2 and 4.5 years (n = 419)	BSID-III test tasks for 2 years, KBIT-II test tasks for 4.5 years at follow-up visit	-	Maternal plasma α, β-carotene, and β-cryptoxanthin levels at delivery	-	-	Postnatal maternal plasma β-cryptoxanthin levels are associated with offspring cognitive and motor skills at 2 years of age (β = 0.18 **, β = 0.16 **), but unrelated to cognitive performance on KBIT-II tasks at age 4.5 years.
Kadam, 2024 [76] ★★ (USA)	2 years of age (n = 38)	Bayley-III Test, Toddler Development™ Screening Test-III, Placental gene expression measurement, Children’s salivary cortisol measurement	-	Maternal plasma lutein: 60.5 ± 8.5 ng/mL Cord plasma lutein: 44.4 ± 5.1 ng/mL	-	Three-day dietary records, Maternal L/Z dietary intake: 1.8 ± 1.8 mg	Maternal lutein and zeaxanthin intake during pregnancy was associated with improved cognitive β = 0.003 **) and language scoring (β = 0.002 *) in children at the age of 2.
Mulder, 2014 [69] ★ (Canada)	Age: 5–6 years (n = 160)	KABC-II-IQ tasks, TONI test PPVT-IV: Peabody Picture Vocabulary Test-4, simultaneous processing, mental processing, learning ability	-	Plasma lutein levels: 0.26 ± 0.11 (0.11–0.53)	-	Dietary FFQ, 24 h dietary records mean dietary intake: 2130 (±2125) mcg/dL	Plasma lutein was associated with dietary intake of lutein (r = 0.479 ***) but was not related to assessed cognitive tasks. Participants’ history of human milk consumption for more than 3 months showed a significant difference with PPVT tasks (*p* = 0·037) and the simultaneous processing scale (*p* = 0·034).
Mahmassani, 2022 [67] ★★ (USA)	Age: 3–11 years (n = 1378)	In 3–6 years: PPVT-III, WRAVMA: Visual Motor Abilities; For 6–11 years, (KBIT), WRAVMA drawing subset: memory and learning, BRIEF, Strengths and Difficulties Questionnaire.	-	-	-	Child’s dietary intake of lutein and zeaxanthin using FFQ: 1.0 (0.4) mg	Higher lutein and zeaxanthin intake is associated with receptive vocabulary on PPVT-III tasks and executive functions. No association was observed between lutein and zeaxanthin intake and any other cognitive or behavioral testing.
Parekh R, 2024 [74] ★★★ (India)	5–12 years (n = 60)	Serum BDNF levels, Cognitive Function Assessment: CFF, VAS, CSHQ-A, Creyo Health Cognitive tests—attention, focus, episodic memory, learning, visuospatial working memory, and processing speed	HFP (Macular Metrics, LLC)	Serum lutein and zeaxanthin	-	-	Mean MPOD improved by 25% on supplementation for 6 months. Improvement in serum lutein and zeaxanthin and BDNF levels, Visual Processing Speed (CFF testing), Visual function, eye strain, and MPOD upon the 6 months LZ (10 + 2 mg) supplementation compared to placebo groups (*p* < 0.05).
Saint, 2018 [71] ★ (USA)	7–13 years (n = 51)	Woodcock–Johnson III test	HFP (Mean MPOD: 0.48 (0.17) [0.19–0.82])	-	-	-	MPOD is weakly related to brief intellectual ability (r = 0.268 *), executive processes (r= 0.288 *), spatial relations(r = 0.299 *), and visual/auditory learning (r = 0.236, *p* ≤ 0.1).
Barnett, 2018 [77] ★ (USA)	8–9 years (n = 56)	IQ assessment: Woodcock–Johnson test Academic Performance assessment: KTEA-II test	cHFP (Mean MPOD: 0.64 ± 0.03)	-	-	Child’s dietary intake (3-day dietary record) Mean LZ intake: 806.6 ± 63.0	MPOD was positively associated with academic achievement (r = 0.4 **). Children’s dietary lutein and zeaxanthin intake were associated with written language performance (r = 0.53 **). Academic achievement was significantly associated with VO_2_max (r = 0.26 **), fat-free massVO_2_max (r = 0.26 *), Intelligence Quotient (r = 0.62 **), and BMI (r = −0.37 **).
Walk, 2017 [70] ★ (USA)	8–10 years (n = 49)	KTEA-II—Academic achievement, Modified flanker task with EEG recording	cHFP (Mean MPOD: 0.61 ± 0.03)	-	-	-	The cognitive processing P3 component in ERP during the modified flanker test for incongruent task was inversely associated with higher MPOD (r= −0.30 *). There were no significant differences in KTEA-II scores between the high and low MPOD quartile groups.
Hassevoort, 2017 [72] ★ (USA)	8–11 (8.8 ± 0.11) (n = 40)	IQ test: Woodcock–Johnson (WJ) brief intelligence assessment standard score, computerized spatial reconstruction task for hippocampal relational memory	cHFP Mean MPOD: 0.66 ± 0.03	-	-	-	MPOD was negatively associated with relational memory error metrics (r= −0.388 **). Bivariate correlations show adiposity was inversely related to IQ (r = −0.460 **) and positively related to relational memory measures misplacement r =0.272 *, edge resizing r =0.281 * and swaps r = 0.329 *.
Rosok, 2025 [78] ★ (USA)	7–13 years (n = 47)	Brain structural and activity assessed using MRI and fMRI at rest and during relational memory tasks	cHFP	-	Veggie Meter	Seven-day diet records: lutein and zeaxanthin intake = 0.72 ± 0.54 mg/d	MPOD was inversely related to the volume of optic chiasms (β = −0.34 **), while skin carotenoid levels were positively associated with inferior temporal white matter volume (β = 0.23 **). MPOD and skin carotenoids were not associated with resting-state global functional connectivity network measures. Dietary lutein and zeaxanthin intake were positively associated with skin carotenoids but not with MPOD.
Rosok, 2022 [73] ★ (USA)	7–12 years (n = 106)	Woodcock–Johnson IV	-	-	Veggie Meter	Block Food Frequency Questionnaire	Skin carotenoid was positively related to quantitates reasoning and broad math skills (applied problems, calculation, math facts, fluency) (r = 0.24 *; r = 0.225 *). Skin carotenoids were positively associated with dietary carotenoids and inversely correlated with body fat%.
Yen, 2022 [68] ★ (USA)	4–5 years (n = 51)	WJ—ECADT-IV for Early Cognitive Academic Development Test	-	-	Veggie Meter	Dietary Intake (7-day record- HEI Index)	Skin carotenoid levels were associated with general intellectual ability, expressive language, and academic skills.
Liu, 2021 [61] ★ (USA)	7–12 years (n = 81)	-	cHFP Mean MPOD: 0.56 ± 0.2	-	Veggie Meter	Seven-day dietary records Mean total carotenoid intake 6.5 ± 0.4 mg, Mean lutein and zeaxanthin intake- 0.8 ± 0.07 mg	Total carotenoid intake was positively associated with skin carotenoid scores (r = 0.25 *) and negatively associated with weight status (BMI percentile, %FAT, VAT) (r = −0.4 **). Children with a history of breastfeeding for <5 months showed lower skin carotenoid scores than those breastfed for ≥5 months.
Aguila, 2014 [79] ★ (USA)	5–7 years (n = 45)	-	-	Carotenoid serum level: 0.59 ± 0.05	Bio photonic Scanner	24 h recalls FFQ Mean total carotenoid intake:1.9 ± 0.21 mg	Multivariate regression model showed total carotenoids measured by resonance Raman spectroscopy were positively associated with total carotenoid intake (R^2^ = 0.25 **). Serum carotenoid levels were correlated with skin carotenoid measured by resonance Raman spectroscopy (R^2^ = 0.62 ***).
Liu, 2021 [60] ★ (USA)	7–9 years (n = 134)	-	cHFP Mean MPOD: 0.57 ± 0.23	-	-	Three-day dietary records Mean LZ intake: 1.17 ± 2.15 (0.07 to 19.2 mg)	MPOD was associated with genetic confounders identified from saliva samples, *BCO1*-T allele, and *CD36*-T, C allele. MPOD was not associated with weight status and dietary intake.
Cannavale, 2023 [80] ★ (USA)	7–12 years (n = 37)	-	cHFP Mean MPOD: 0.56 ± 0.23	-	-	Seven-day dietary record, mean LZ intake: 146.9–2596.6 (752.7 ± 508.0)	MPOD showed a negative association with serum C-reactive protein levels in children.
Cannavale, 2023 [81] ★ (USA)	7–13 years (n = 181)	-	cHFP, mean MPOD: 0.56 ± 0.22	-	Veggie Meter	Mean Carotenoids Intake: 5.03 mg/1000 kcal Mean LZ Intake: 0.81 mg/1000 kcal	Skin carotenoid score was associated with total carotenoid intake and BMI%. Lutein and zeaxanthin intake were associated with MPOD (stdβ = 0.27 **). No association observed between skin carotenoid and MPOD.
Marta-C, 2024 [82] ★ (Spain)	7–13 years (n = 27)	-	HFP, Mean MPOD: 0.45 ± 0.14 (0.19–0.91)	-	-	Dietary Intake: KIDMED Questionnaire	MPOD levels were positively associated with daily fruit or fruit juice consumption in children (*p* = 0.034), but not with total KIDMED dietary questionnaire scores, age, BMI, and LED screen exposure of more than five hours per day.

★ Observational, Cross-sectional, ★★ Prospective longitudinal/Cohort, ★★★ Randomized controlled trial. * = *p* < 0.05, ** = *p* < 0.01 and *** = *p* < 0.001. Abbreviations: LZ: Lutein and Zeaxanthin. ERP: Event Related Potential test. MMP: Mismatch Negativity responses. VEP: Visual Evoked Potential test. BRIEF: Behavior Rating Inventory of Executive Function, KABC: Kaufman Assessment Battery for Children. KBIT-II Kaufman Brief Intelligence Test, Second Edition. KTEA II: Kaufman Test of Educational Achievement, Second Edition. PPVT: Peabody Picture Vocabulary Test. TONI-3: Test Nonverbal Intelligence, Third Edition: assesses aptitude, abstract reasoning and problem solving. WRAVMA: Wide Range Assessment of Visual Motor Abilities. WJ-ECAD: Woodcock–Johnson Early Cognitive and Academic Development Test, composite standard scores were obtained for Brief Intellectual Ability, Verbal Ability, Cognitive Efficiency, Processing Speed, and Executive Process.

## 5. Ocular Biometry and Macular Pigment Optical Density

Ocular biometry parameters, axial length, refractive error, corneal curvature, retinal thickness, and choroidal thickness vary with age and may vary in several pathological conditions of the eye. Various studies have found that the level of retinal carotenoids measured at the macula serves as a biomarker, and the antioxidant capacity of the retina as a whole may play a role in the pathogenesis of various retinal disorders like macular degeneration, diabetic retinopathy, and glaucoma [56,83,84,85]. This section will highlight the literature on ocular biometry and macular pigment density in the pediatric population.

### Refractive Error, Its Progression and Macular Pigment Distribution

Myopia is the most common refractive error in children. Eyes with myopia have longer axial length than eyes with emmetropia, and progression of this condition is significant in early developmental years, with progressive elongation of the eyes’ axial length. This could cause pathological changes and is known to cause maculopathy and increased risk of glaucoma [86]. Table 2 provides a summary of MPOD levels in children. Zheng et al. reported the association of cHFP-assessed MPOD with foveal thickness in Chinese children with a mild/moderate degree of myopia (MFT, r = −0.66, CFT, r = 0.67) [87]. However, in mild refractive error cases, MPOD measured through the objective method on a Visucam 200 was not significantly associated with ocular biometric parameters, including axial length, degree of refractive error, corneal curvature, and age [88].

Li et al. [89] evaluated in a randomized controlled trial the benefits of lutein esters in children aged eight to twelve years and found that supplementation led to effectively mitigating sub-foveal and temporal choroidal thinning, which are known biomarkers of increased myopia. These lutein and zeaxanthin intervention studies suggest that MPOD levels in children and adults can be improved with active lutein supplementation for three to six months in a high degree of myopia and in eyes with large myopic pathological changes of the posterior segment.

In adults, MPOD showed a positive association with retinal thickness parameters [90], with notably lower levels in cases of pathological myopia [91]. Adding to this, MPOD appears to be a modifiable biomarker, as a few studies have also reported improvement in MPOD following lutein supplementation in individuals with high or pathological myopia [92,93]. Although there is increasing evidence of possible benefits of carotenoids in the prevention of myopia and its progression, much more research will be needed before this can become a form of adjunctive therapy.

**Table 2:** Literature summary of Macular Pigment Optical Density assessment among children with refractive error.

Author, Year, Country (MPOD Technique)	Age and Sample Size, Refractive Error Range	Mean MPOD	Study Results
Zheng, 2013 [87] China (cHFP; Macular Metrics II Densitometer)	6–12 years (n = 94) Spherical equivalent range: −5.5 to +4.37 D	0.56 ± 0.25	MPOD showed no significant association with age, BMI, IOP, refractive error, MFT, or CFT. MPOD was inversely correlated to axial length. In the myopia refractive group, there was a negative relationship between MPOD and MFT (R = −0.66, *p* = 0.028) and a positive association between MPOD and CFT (R = 0.67, *p* = 0.025).
Liu, 2022 [88] China (Visucam 224)	6–14 years (n = 902) Spherical equivalent range: −1.26 ± 1.02 D	0.28 ± 0.08	MPOD was not associated with ocular biometric parameters (i.e., axial length, refractive error, corneal curvature) and age. Axial length, corneal curvature, and refractive error effectively increase with age.
Wang, 2022 [94], China. (Visucam-200)	6–10 years (n = 40)	Hyperopic Anisometropic amblyopia: 0.12 ± 0.03 d.u. Fellow eye: 0.13 ± 0.04 d.u	Hyperopic anisometropic amblyopic eyes reported marginally low mean MPOD compared to fellow eyes.
Erkan Turan, 2018 [95], China. (MPS II) Mean age:	9 years (n = 54 Healthy + 41 Strabismus)	Healthy eyes: 0.23 ± 0.25 non-preferred eye of strabismic children: 0.25 ± 0.27 Preferred eye of strabismic children: 0.43 ± 0.34	Preferred eye of strabismic children tends to have more macular pigment deposition compared to non-fixating eye and healthy children.

MPOD, macular pigment optical density; AL, axial length; BCVA, best-corrected visual acuity; BMI, body mass index; IOP, intraocular pressure; MFT, mean foveal thickness; CFT, central foveal thickness; HFP, heterochromatic flicker photometry; GCL, ganglion cell layer; IPL, inner plexiform layer; ONL, outer nuclear layer; ERG, electroretinography; SER, spherical equivalent refraction.

Macular pigment optical density measures in eyes with low to moderate degrees of hyperopia have been found likely to resemble the results of emmetropic eyes [96,97]. An uncorrected or high degree of ametropia in early life may result in anisometropic or strabismic amblyopia [98,99]. Investigations by Kaya et al. [97] and Wang et al. [94] indicated that the MPOD concentration is relatively lower in eyes with hyperopic-anisometropic amblyopia compared to the fellow healthy eye of the participant and the controlled emmetropic cohort of the study group. The MPOD was assessed in these studies using a Visucam-200 which is based on the principles of single wavelength fundus reflectometry and objectively measures the absorption of blue wavelength to calculate MPOD. The study results indicate that the central macular thickness and visual acuity were inversely associated with MPOD levels. In the cases of children with strabismus, non-fixating eyes had lower MPOD compared to fellow fixating eyes; MPOD was measured using the MPS-II technique [95]. Further research is warranted in strabismic eyes to confirm this finding and the mechanisms that dictate the level of MPOD in eyes with varying refractive errors need to be explained. It will also be interesting to evaluate the benefits of standalone vision therapy or when vision therapy is combined with additional interventions with diet or supplementation of carotenoids to evaluate benefits and improvements in visual function in refractive and strabismic amblyopia.

## 6. Carotenoids for Congenital Vascular and Inherited Retinal Disease

### 6.1. Carotenoids and Retinopathy of Prematurity

Retinopathy of prematurity (ROP) is a progressive congenital vascular retinal condition commonly seen in premature infants. This condition usually presents between the fourth and tenth weeks after premature birth and progresses until around 41 weeks from postmenstrual age (PMA) [100,101,102]. However, this condition is rare in full-term babies and is called Retinopathy of Prematurity like Retinopathy (ROPLR) [103]. Carotenoids are protective against retinal endothelial cell injury, and potential application at a premature age may help in protecting retinal cells in congenital retinal vascular conditions.

Human milk and infant plasma contain the highest lutein and beta-carotene content of all carotenoids, depending on the ethnic region and maternal dietary habits [8,9]. Additionally, a recent study from Israel observed that human milk lutein concentrations do not vary between mothers of non-ROP and ROP children [104]. However, macular pigments are very low in the retinas of premature babies [39,40]. In Romagnoli et al. (2011), Dani et al. (2012), Rubin et al. (2012), and Manzoni et al. (2013) [52,105,106,107], trials in premature infants reported that macular carotenoid supplementation did not reduce the incidence rate of ROP significantly. However, carotenoid supplementation had mixed results on decreasing progression of ROP from early to late stages, with some studies showing potential benefits. The mixed outcomes may in part be due to lower doses of lutein and zeaxanthin in the supplementation trials. The trials used a relatively low dosage of lutein and zeaxanthin, i.e., 0.14 mg + 0.006 mg/day or 0.5 + 0.02 mg/kg/day orally, and about 211 mg lutein/liter in fortified formula, which may have been inadequate to elucidate clinical benefits [108]. Rubin et al. observed improved rod cell function relative to lutein plasma levels and reduced CRP levels followed by lutein supplementation [52]. In a mouse model of ROP, a dose of 0.2 mg/kg/day of lutein for 5 days pointed to its significant effect in promoting endothelial cell growth and reducing blood vessel leakage [109]. Vitamin A has been shown to considerably decrease vascular endothelial growth factor (VEGF) in ROP children [110]; however, it should be noted that a high dosage of vitamin A may pose a risk of toxicity, but lutein and zeaxanthin are graded as generally recognized as safe (GRAS) supplements, even at high doses. A recent BCO2-/- mouse model study showed that prenatal exposure to macular carotenoids reduces ROP-like damage [111]. The study investigated varying dosages of lutein and zeaxanthin for different durations, and they noted that zeaxanthin had a more pronounced effect on protecting against retinal vascular damage. Therefore, it may be interesting to explore the additional benefits of lutein and zeaxanthin at higher doses or in a combination of both infants with ROP and those at risk of ROP.

In human trials, prenatal carotenoid supplementation with 10 mg lutein and 2 mg zeaxanthin in low-risk pregnant women (i.e., no pre-existing medical condition, singleton pregnancy, no history of complicated pregnancies, normal BMI range) resulted in early maturation of the macular region and a 20% higher concentration of MPOD in healthy newborns compared with controls [41]. There is still a limited number of studies to make clinical recommendations, and lutein and zeaxanthin supplementation trials in women with high-risk pregnancies (i.e., pre-existing health condition, adolescent pregnancy, multifetal pregnancy, history of pre-eclampsia, prior history of intrauterine growth restriction, premature delivery, drug abuse, etc.) would be equally important to investigate in the future as they have comparatively increased oxidative stress and inflammation.

The current evidence regarding macular carotenoid supplementation and ROP prevention remains inconclusive. One can hypothesize that greater intake of carotenoids in the infants with ROP may lead to better outcomes clinically. These are based on the following reasons: (1) the published literature has considered a relatively lower dose of carotenoids, (2) the safety profile of the dietary macular carotenoids is high and at a low risk overall, and (3) prior studies have shown that greater maternal intake of carotenoids may lead to better maturity in the retina, particularly the foveal region. Future research is crucial to define optimal dosage levels, effective modes of supplementation, duration of carotenoid exposure, derivative sources of supplements to improve carotenoid levels, and causes of its attenuation in the retina. Anatomically, zeaxanthin is concentrated in great amounts in the central macular region compared to lutein, and hence the optimal ratios of zeaxanthin-to-lutein will also need investigating [112].

### 6.2. Role of Carotenoids in Pediatric-Onset Inherited Retinal Degenerative Diseases

At a younger age, congenital inherited retinal diseases (IRDs) progress rapidly in severe forms relative to later years of life. For decades, nutrition has been an integral part of finding a cure for incurable chronic conditions. Today, understanding the molecular pathway of the disease condition and its interaction with antioxidants guides the targeted, specific forms of nutrition recommendations. Historical biochemical studies have found relatively persistent low levels of carotenoids and vitamin A and slightly raised levels of total cholesterol in patients with retinitis pigmentosa for years [113]. Several pieces of literature have studied the role of nutrition in congenital degenerative conditions [114]. Currently, provitamins and synthetic derivatives of carotenoids are under investigation to treat defective molecular pathways in conditions like retinitis pigmentosa (RP), Stargardt disease, and Leber congenital amaurosis.

Berson et al. [115,116] evaluated the use of lutein and vitamin A supplementation in individuals with RP. Of particular note, Berson et al. followed a group of children in a 5-year longitudinal trial and found that an age-adjusted dose of oral vitamin A palmitate (≤15,000 IU/d) led to a slower rate of cone dysfunction and RP disease progression among children [116]. There is a shortage of studies performed in children on the intake of carotenoids like lutein. In their prior work, Benson et al. [115] reported that lutein supplementation of 12 mg/day in combination with vitamin A in non-smokers slowed down the progression of visual field loss.

New pharmacotherapies using disease-specific synthetic carotenoid forms are being explored to repair the impaired visual cycle [117,118]. These have not been explored in children; however, remarkable improvements in visual acuity and visual field were observed in individuals at least 18 years of age when an oral formulation of 40 mg of 9-cis-β-carotene and 9-cis-retinyl-acetate was used for seven days in patients with certain forms of retinitis pigmentosa and Leber congenital amaurosis. C20-D3-vitamin A and 13-cis-retinoic acid oral formulas for Stargardt disease are still under investigation for their safety and efficacy [119]. Other emerging retinal carotenoid regulatory reservoir therapies that target retinal pigment epithelium include RPE 65 targeted gene therapy, which allows regenerating the 11-cis-retinol in the visual cycle in the retinal pigment epithelium. On the other hand, photoprotection through sunglasses and tinted lenses is also reported to prevent light damage by limiting the light-stimulated conversion of 11-cis-retinol to 11-trans retinol. As a result, photoprotection helps in reducing lipofuscin deposition in patients with Stargardt macular dystrophy [120]. Lipofuscin deposition is inversely related to MPOD. The MPOD measure is relatively low among patients with RP, Usher Syndrome, and Stargardt macular dystrophy [121,122,123]. Overall, although there is a body of work in adults that suggests potential improvement with carotenoid vitamin therapy, this will need further validation in children.

## 7. Conclusions

Overall, the evidence synthesized in this scoping review indicates that dietary carotenoids—particularly lutein, zeaxanthin, and β-carotene—are present in maternal serum, human milk, infant serum, and in brain and retinal tissues. Additionally, MPOD, serum carotenoid levels, and non-invasive skin measures serve as useful biomarkers for assessing carotenoid status and have been associated with visual and cognitive performance in children. Observational studies and a small number of supplementation trials report positive associations between higher carotenoid exposure (prenatal, breast milk, or dietary/supplementation) and improved recognition memory, psychomotor outcomes, receptive vocabulary, executive function, and some academic measures, while human milk appears more bioavailable than many fortified formulas. However, the literature remains heterogeneous, largely observational, and limited by variable outcome measures, small sample sizes, and inconsistent dosing. There is a scarcity of data produced by randomized controlled trials in infants and long-term follow-up into later childhood. Mechanistic and clinical signals—benefits for retinal maturation, possible mitigation of myopic changes, and neuroprotective effects in certain retinal diseases—are promising but not yet definitive. Therefore, we conclude that targeted, well-powered longitudinal RCTs are needed to define optimal carotenoid types, doses, timing (prenatal vs. postnatal), and bioavailability strategies (human milk-mimicking formulations), to establish causality, safety thresholds for children, and whether early-life carotenoid exposure yields durable gains in vision and cognition.

## Data Availability

No new data were created or analyzed in this study.

## Supplementary Materials

The following supporting information can be downloaded at: https://www.mdpi.com/article/10.3390/nu18132147/s1, Table S1: Studies included in the scoping review.
