# Supplementary material for: Associations Between Carotenoid Status, Visual Outcomes and Cognitive Metrics in Children: A Scoping Review
Source: Nutrients. 2026 Jul 2;18(13):2147. doi: 10.3390/nu18132147 (PMC13363774; doi:10.3390/nu18132147)
Supplement: Supplementary file 1 [file nutrients-18-02147-s001.zip › nutrients-4362001-supplementary.pdf]

Table S1

Studies included in the scoping review

No	Study design (Author, year) [citation identification number]	Study design
1	Panova et al. (2017) [36]	Observational analytical study
2	Henriksen et al. (2013) [38]	Cross-sectional
3	Bernstein et al. (2013) [39]	Cross-sectional
4	Sasano et al. (2018) [40]	Cross-sectional
5	Addo et al. (2023) [41]	RCT
6	Addo et al. (2024) [42]	RCT
7	Rubin et al. (2012) [52]	RCT
8	Liu et al. (2021) [60]	Cross-sectional
9	Ponce-García et al. (2025) [62]	Cross-sectional
10	Liu, 2021 [61]	Retrospective observation of previous RCT
11	Cheatham and Shepard, 2015 [64]	Observational prospective cohort study
12	Zielinska, 2019 [65]	Observational prospective cohort study
13	Lai, 2020 [66]	Observational prospective cohort study
14	Mahmassani, 2022 [67]	Observational prospective cohort study
15	Yen, 2022 [68]	Cross-sectional
16	Mulder, 2014 [69]	Cross-sectional
17	Liu, 2021 [60]	Cross-sectional
18	Walk, 2017 [70]	Cross-sectional
19	Saint, 2018 [71]	Cross-sectional
20	Hassevoort, 2017 [72]	Cross-sectional
21	Rosok, 2022 [73]	Cross-sectional
22	Parekh R, 2023 [74]	RCT
23	Rosok, 2024 [75]	Cross-sectional
24	Kadam, 2024 [76]	Prospective observational study
25	Barnett, 2018 [77]	Cross-sectional
26	Rosok, 2025 [78]	Cross-sectional
27	Aguila, 2014 [79]	Cross-sectional
28	Cannavale, 2023 [80]	Cross-sectional
29	Cannavale, 2023 [81]	Cross-sectional
30	Marta-C, 2024 [82]	Cross-sectional
31	Zheng, 2013 [87]	Cross-sectional
32	Liu, 2023 [88]	Cross-sectional
33	Li, 2025 [89]	RCT
34	Wang, 2022 [94]	Cross-sectional
35	Erkan Turan, 2018 [95]	Cross-sectional
36	Uretzky, 2024 [104]	Prospective longitudinal observational study

37	Romagnoli, 2011 [105]	RCT
38	Dani, 2012 [106]	RCT
39	Manzoni, 2013 [107]	RCT
40	Berson, 2018 [116]	Nonrandomized retrospective case-control study
